# Comment on Mroczek *et al.* Evaluation of Quality of Life of Those Living near a Wind Farm. *Int. J. Environ. Res. Public Health*, 2015, *12*, 6066–6083

**DOI:** 10.3390/ijerph14020140

**Published:** 2017-02-01

**Authors:** Daniel Shepherd

**Affiliations:** Department of Psychology, Auckland University of Technology, Auckland 0627, New Zealand; daniel.shepherd@aut.ac.nz; Tel.: +64-9-921-9999 (ext. 7238); Fax: +64-9-921-9780

**Keywords:** noise, wind turbines, health

## Abstract

An article published in the *International Journal of Environmental Research and Public Health* indicated that, far from degrading health, noise from wind turbines may actually be associated with positive health outcomes. Such a finding is counter to that reported elsewhere for general and wind turbine noise. This Commentary sets out to explore alternative explanations of these differences.

## 1. Introduction

The article published in the *International Journal of Environmental Research and Public Health* by Mroczek et al. [1] is remarkable for a number reasons, and invites further comment. The data presented here is not new, having been previously published almost in replicate [2], and so the findings detailed in this latest version of the data have already been well-considered by the noise and health community. Regrettably, many of the shortcomings of the original paper have been exported to its reincarnation. Certain omissions make it difficult to assess the quality of the study, and I feel a closer look at these omissions are warranted.

Omission One: In support of their statement that “No scientific evidence has been found so far in favor of the influence of turbines (in particular, of their noise) on health” (p. 6067) the authors cite two reviews [3,4]. The first, out-dated and widely derided [5], was undertaken by a pharmacologist. The second study [4] states “The quality and quantity of the available evidence was limited” (p. 17), and I would like to paraphrase that quote and say that the quality, but unfortunately not the quantity, of said review is also limited. A more recent and more competently undertaken review is available from the University of Oxford [6]. They review the studies omitted from Mroczek’s introduction [1], those which arguably present scientific evidence that wind turbine noise can impact health in the same way that road and aviation noise can.

Omission Two: While Mroczek et al. [1] focus on dispositional factors such as attitudes and “nocebo”, they unfortunately fail to frame the study in the wider context of the Polish wind industry. Specifically, they omitted the findings by Poland’s Supreme Audit Office [7], who reported widespread bribery and corruption in Poland’s wind farm construction decision making processes. Furthermore, the Supreme Audit Office report indicates that, through untoward payments to local officials, up to 80% of wind farms were erected without consideration to environmental, health, or safety matters. These contextual factors are needed when considering the author’s [1] subsequent analyses and commentary.

Omission Three: The means for the SF-36 Scale were not reported for the groups of interest. This makes it very difficult to determine whether the differences between the groups (i.e., effect sizes) were substantial or not. This is important when one has large sample sizes such as those reported in the Mroczek et al. study (*n* = 1277), as very small and clinically insignificant differences may in fact reach statistical significance. In fact, the means reported in their Table 4 (p. 6074) are not relevant to their research objectives, and while the authors laud the “availability of normative data” (p. 6070), they do not compare their data to these available norms, nor could I access any through the internet. In fact, what rural data I could find [8] were substantially at odds with those reported in [1]. Furthermore, their averaging of the eight SF-36 subscales (p. 6072) is not sanctioned by the originator of the SF-36 [9], and has no precedence in the literature.

Omission Four: There is not enough detail in the analysis section to understand why the authors did what they did, and how they did it. This not only makes interpreting the results of the analyses difficult, but can impede replication.

Omission Five: Their novel finding, that far from being detrimental to health, living in the vicinity of wind turbines may actually promote health, diverges from research reported from countries as diverse as Australia, Canada, Denmark, Holland, Iran, Japan, New Zealand and Scandinavia (see [6]). In their discussion they have omitted to explore why their findings depart substantially from these reports, and they have likewise to propose a realistic causal mechanism. That said, their conclusion that “The available results imply that wind turbines, when located at a proper distance, do not exert any negative effects on human health” (p. 6080) could not be more correct.

Omission Six: The world of noise research is a small and mostly collegial one, and it is not always easy to keep personal matters private. That the first author is married to an owner of E.P.A. holdings, a firm which specializes in wind turbines, is of concern. The fact that this relationship was not disclosed in the section dedicated to ‘Conflicts of Interest’ is indeed the most astonishing omission of all.

## 2. Conclusions

Taken together, these omissions detract from what otherwise could have been a very important and influential paper.
